# Deciphering the Complexity in the Rotational Spectrum of Deuterated Ethylene Glycol

**DOI:** 10.1021/acsearthspacechem.5c00067

**Published:** 2025-04-29

**Authors:** Jordan A. Claus, Mattia Melosso, Agathe Maillard, Luca Bizzocchi, Vincenzo Barone, Cristina Puzzarini

**Affiliations:** † Dipartimento di Chimica “Giacomo Ciamician”, Università di Bologna, Via F. Selmi 2, 40126 Bologna, Italy; ‡ INSTM, 50121 Firenze, Italy

**Keywords:** ethylene glycol, complex organic molecules, interstellar medium, millimeter-wave, rotational spectroscopy

## Abstract

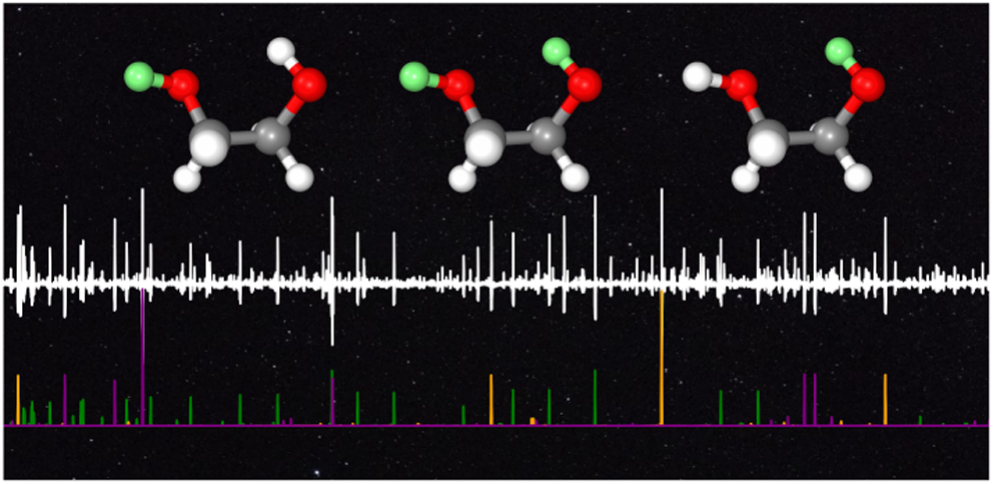

Ethylene glycol (CH_2_OH–CH_2_OH) is an abundant “complex organic molecule” (COM) detected in different astronomical objects, but the steps of its interstellar synthesis are not yet fully understood. In this respect, the observation of deuterated isotopologues could offer insights into its formation mechanism as well as into its chemical evolution in space. Such observations, however, require detailed spectroscopic knowledge of their rotational features. Here, we present an extensive analysis of the rotational spectrum of oxygen-deuterated ethylene glycol, including the singly and doubly deuterated forms. The new measurements, carried out between 75 and 450 GHz, significantly expand the spectroscopic knowledge of the *aGg*′ conformers of the CH_2_OH–CH_2_OD, CH_2_OD–CH_2_OH, and CH_2_OD–CH_2_OD species. We also report, for the first time, the laboratory identification of the *gGg*′ conformers of the two mono-deuterated species. Our results reveal previously unobserved perturbations arising from the interaction between CH_2_OH–CH_2_OD and CH_2_OD–CH_2_OH, which has been modeled by including Coriolis coupling and Fermi constants in the Hamiltonian and allowed the accurate determination of the energy difference among them. Additionally, we observed significant anomalies in the spectrum of the doubly deuterated species, which seem to be caused by accidental degeneracies between the levels of the two tunneling substates. Despite the complexity and difficulties, the improved spectroscopic parameters derived from our analyses provide a solid base for future interstellar searches of deuterated ethylene glycol, enhancing our understanding of the evolution of COMs in the interstellar medium.

## 1 Introduction

Ethylene glycol (CH_2_OH–CH_2_OH, also known as 1,2-ethanediol) and 1,2-ethenediol (CHOH=CHOH) are the only diols discovered in the interstellar medium (ISM) to date. While the latter has only been detected in its *Z* form in the G+0.693-0.027 molecular cloud,^1^ the former has been observed towards a variety of astronomical sources, including Class 0 protostars,^2,3^ star-forming regions,^4−6^ hot-cores,^7,8^ and comets.^9,10^ Ethylene glycol belongs to the family of the so-called “Complex Organic Molecules” (COMs)^11^ and, being the reduced form of glycolaldehyde (CHO–CH_2_OH), is indirectly involved in those processes that lead to the formation of more complex sugars, such as the simplest aldose, glyceraldehyde (CHO–CH_2_OH–CH_2_OH), and the simplest sugar alcohol, glycerol (CH_2_OH–CH_2_OH–CH_2_OH).^12,13^

A number of experimental, theoretical, and observational studies have been devoted to understand the ethylene glycol formation in the ISM. It has been shown that ethylene glycol, together with glycolaldehyde, can be formed by subsequent hydrogenation of CO on interstellar ice analogues without the aid of external energetic sources.^14^ In this process, HCO and CH_2_OH radicals are observed as key intermediates.^15^ In addition, the direct hydrogenation of glycolaldehyde to ethylene glycol has also been studied in the laboratory and found to be efficient even at the low temperatures of dark clouds.^16^ All of these studies clearly demonstrated that ethylene glycol can be efficiently formed through dust-grain chemistry even in the early stages of star formation, that is, the pre-stellar phase. These laboratory findings are strongly supported by astronomical observations of ethylene glycol and glycolaldehyde,^17,18^ which indicate (i) a common origin of these two COMs, (ii) a correlation between their ratio and the source luminosity, and (iii) similar spatial distributions in the Sgr B2 region.^4,19^ Despite these studies, not all of the intermediate steps of the interstellar ethylene glycol synthesis have been fully understood. For example, it is unclear whether the main formation pathway is the hydrogenation of glycolaldehyde to ethylene glycol or the radical-radical recombination of two CH_2_OH units on the grain surface.^20^ In this context, the observation of deuterated isotopologues of ethylene glycol in the ISM and the determination of their abundance ratio with respect to deuterated glycolaldehyde can provide useful insights into their interstellar chemistry.^3^ Indeed, the replacement of hydrogen by deuterium affects the rate constant of the hydrogenation process, which is reduced by about 1 order of magnitude due to the lower surface mobility of D with respect to H.^14^ In turn, such a change alters the branching ratio of the reaction channels in different ways, thus favoring the formation of specific isotopologues. In addition, the observation of deuterium fractionation in ethylene glycol would provide further information on the evolutionary stage during which ethylene glycol is formed and can help us understand whether or not the parent species emission suffers from optical thickness.^3^ For all of these reasons, to complement our recent spectroscopic characterization of deuterated glycolaldehyde and (*Z*)-1,2-ethenediol,^21^ we decided to deeply investigate the rotational spectrum of deuterated ethylene glycol. Here, we focus on deuteration of the hydroxyl groups, but investigation of the carbon deuterated species is also important and deserves to be addressed in the future.

Previous spectroscopic studies on the parent species of ethylene glycol include the measurement of the rotational spectrum and its analysis for the two most stable conformers, namely, *aGg*′ and *gGg*′, from the microwave^22,23^ to the sub-millimeter-wave region.^24−26^ For the deuterated isotopologues, either oxygen- or carbon-substituted species, laboratory data are much more limited. Specifically, for the singly and doubly O-deuterated forms, only the *aGg*′ conformer has been investigated, the work being limited to the microwave domain (18-50 GHz).^27,28^ For the doubly deuterated CH_2_OH–CD_2_OH variant, instead, both the *aGg*′ and *gGg*′ forms have been studied, again only at low frequencies (below 41 GHz).^29^ In this respect, it should be noticed that most of the deuterated COMs detected in recent years (see, e.g., refs (30−32)) have been observed in spectral line surveys of IRAS 16293-2422 taken with ALMA in Band 3 (90–104 GHz)^33^ and in Band 7 (329–363 GHz).^3^ Since extrapolation of low-frequency experimental data to high-frequency spectral predictions often results in a significant shift, which prevents the molecular identification,^26,34^ it is essential to extend measurements and analysis to the millimeter/sub-millimeter-wave region. This is especially important whenever the molecular spectrum is affected by large amplitude motions, as in the case of ethylene glycol.

In this work, we report a thorough analysis of the rotational spectra of singly and doubly O-deuterated ethylene glycol based on our new measurements between 75 and 450 GHz. We have greatly improved the dataset available for the *aGg*′ conformer of the CH_2_OH–CH_2_OD, CH_2_OD–CH_2_OH, and CH_2_OD–CH_2_OD species, and we successfully identified the *gGg*′ conformers of the mono-deuterated species for the first time.

The manuscript is organized as follows. In the next section, the experimental details are summarized. Subsequently, Section 3 addresses the strategy pursued, the difficulties encountered, and the results obtained in the analysis of the rotational spectra of the mono- and bideuterated species of ethylene glycol, considering both the *aGg*′ and *gGg*′ conformers. This section is followed by a thorough discussion about the assignment problems and interactions characterizing the rotational spectra of these molecules. Finally, concluding remarks are provided.

## 2 Experimental Details

Ethylene glycol (anhydrous, 99.8% purity) and heavy water (D_2_O, 99.9% of D atom) were purchased from Merck and used without further purification. The sample of deuterated ethylene glycol was prepared by mixing ethylene glycol and heavy water in a 1:1 molar ratio. This procedure also forms HDO and H_2_O, which were then removed together with the remaining D_2_O by pumping on the sample. The mixture resulting from this procedure mainly contains singly and doubly O-deuterated ethylene glycol and will be denoted as “deuterated ethylene glycol” (or, simply, “deuterated sample”) in the following.

The rotational spectrum of deuterated ethylene glycol was recorded between 75 and 450 GHz using a W-band Signal Generator Extension module (Mini SGX, Virginia Diodes, Inc., WR10SGX-M) as a millimeter-wave radiation source. The system is based on a centimeter-wave signal generator (0.25–20 GHz, Keysight Technologies, N5173B) whose frequency is multiplied by 6 and amplified by the SGX module to cover the 75–110 GHz frequency interval. Higher frequencies are obtained by a cascade of active and passive multipliers (WR5.1x2 for 140–220 GHz, WR3.4x3 for 220–330 GHz, WR2.2x2 for 330–500 GHz, Virginia Diodes, Inc.) based on planar GaAs Schottky diode technology. The emitted radiation was sine-wave modulated at a frequency of 16.67 kHz, with a modulation depth varying between 100 and 300 kHz according to the Doppler linewidth of the target signals. Both frequency and phase stabilizations are achieved by referencing the N5173B synthesizer to a 10 MHz rubidium atomic clock (Stanford Research Systems, FS725).

Experiments were conducted by filling the free-space glass absorption cell (3.25 m long, 5 cm diameter) with deuterated ethylene glycol vapor in flow condition at a pressure of about 7.5 mTorr. The radiation source is collimated into the cell by using a parabolic mirror (ThorLabs, MPD269-M03) and focused via a second parabolic mirror on the detector. Two high-density polyethylene windows are used to seal the cell while allowing radiation to pass through. The pressure inside the cell is monitored by a pressure gauge (Leybold Vacuum, CERAVAC CTR 100N). The cell is maintained under a vacuum by a pumping system composed of a diffusion oil pump and a primary pump.

The molecular signal was detected by several zero-bias Schottky-barrier diodes (Virginia Diodes, Inc.) working in different bands: WR10ZBD (75–110 GHz), WR5.1ZBD (140–220 GHz), WR3.4ZBD (220–330 GHz), and WR2.2ZBD (330–500 GHz). The detector signal was filtered and amplified by a low-noise preamplifier (Stanford Research Systems, SR560) and then demodulated using a lock-in amplifier (Stanford Research Systems, SR830). Subsequently, the resulting DC signal is transmitted to the computer and digitized via an analog-to-digital conversion card (National Instruments, SCB-68A). Recorded spectra are observed as the second derivative of the actual absorption profiles due to the use of phase-sensitive detection at twice the modulation frequency (2*f*). Measurement uncertainties are estimated to range between 20 and 40 kHz depending on the linewidth and signal-to-noise ratio (SNR).

## 3 Analysis of the Spectrum

Out of the ten theoretically possible conformers of ethylene glycol, only the two lowest-energy *gauche* structures, *aGg*′^22,24,26^ and *gGg*′^25^ (see Figure 1 of ref (26) for a graphical representation of their structure), both exhibiting intramolecular hydrogen bonding, have been extensively studied in the laboratory and characterized in the (sub)millimeter-wave region. To date, they remain the only conformers observed. In this nomenclature, small letters refer to the orientation of the two hydroxyl groups (*a* standing for *anti* and *g* for *gauche*), while the capital letter denotes the (*gauche*) orientation of the O–C–C–O backbone. Both conformers are nearly prolate asymmetric rotors with κ = −0.818 and *κ* = −0.822 for *aGg*′ and *gGg*′, respectively. The *aGg*′ conformer possesses a strong dipole moment component along the *a*-axis (μ*_a_* = 2.080(3) D), a medium one along the *b*-axis (μ_*b*_ = 0.936(1) D), and a small component along the *c*-axis (μ_*c*_ = −0.47(1) D).^22^ The *gGg*′ conformer, instead, possesses medium dipole moment components along all three principal axes (μ_*a*_ = 1.30 D, μ_*b*_ = 1.37 D, μ_*c*_ = 1.42 D).^25^

For O-deuterated isotopologues, three possible isotopic species can be considered: (i) the two mono-deuterated species CH_2_OD–CH_2_OH and CH_2_OH–CH_2_OD, hereafter simply referred to as ODOH and OHOD, respectively; (ii) the doubly deuterated CH_2_OD–CH_2_OD isotopologue, hereafter simply denoted as ODOD.

**Figure 1:**
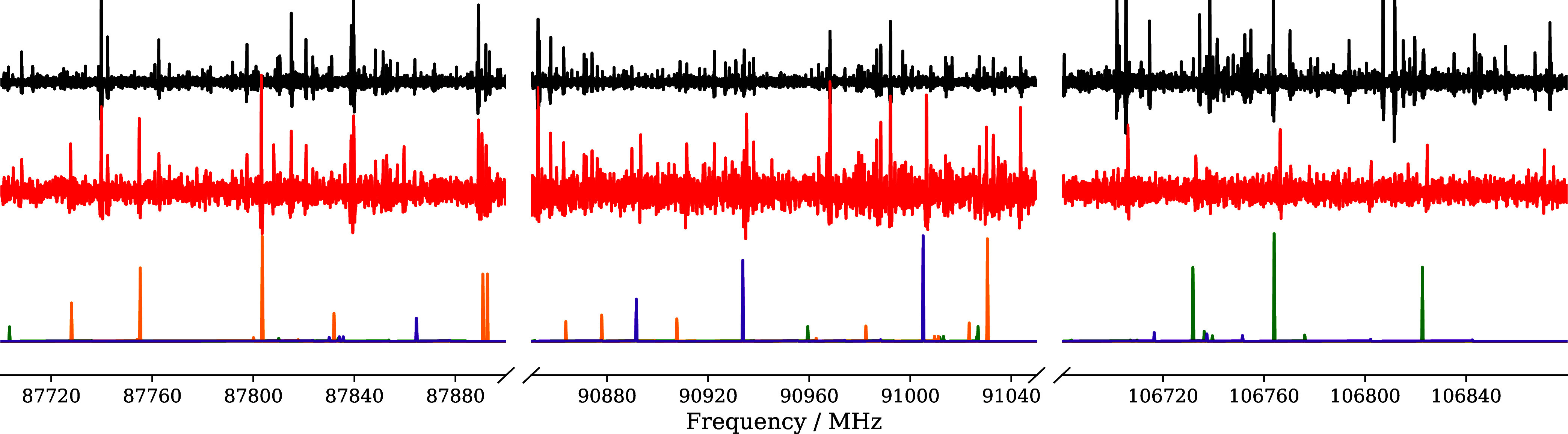
Portions of the millimeter-wave spectrum between 75 and 110 GHz. Top: The black and red traces are the recorded spectra using pure ethylene glycol and our deuterated sample, respectively. Bottom: the stick-spectra simulations of the *aGg*′ conformers are based on the low-frequency data,^22,27,28^ with ODOD in green, OHOD in orange, and ODOH in purple (for the acronym explanation, see text).

Low-frequency data from previous investigations of the *aGg*′ conformers were re-analyzed using SPFIT^35^ and PGOPHER,^36^ and the spectroscopic parameters derived in this way were used for simulating the spectra of all the three deuterated species at higher frequency. In the 75–110 GHz range, transitions were mostly searched for line-by-line within a 10 MHz window around the predicted frequencies. The recorded spectra were then compared with our simulations and a reference spectrum of pure (nondeuterated) ethylene glycol, thus enabling the rapid identification of those transitions belonging to the deuterated species (Figure 1). The assignment and the analysis of newly recorded transitions, most of which were of *a*-type, allowed us to refine all spectroscopic parameters and improve the accuracy of spectral predictions at higher frequencies. In an iterative fashion, we recorded and analyzed the frequency regions associated with the second, third, and fourth harmonics of our spectrometer. The 150–220 GHz frequency range was mostly investigated line-by-line, whereas the spectra were recorded continuously in the 220–330 GHz and 330–450 GHz ranges. In addition to the SPFIT/SPCAT package and PGOPHER, the LLWP^37^ visual tool was used to facilitate the spectral assignment.

### 3.1 Mono-Deuterated Species—*aGg*′ Conformation

At the beginning of our study, and in line with the previous spectroscopic work,^28^ the *aGg*′ conformers of OHOD and ODOH were treated as two distinct species, since the isotopic substitution makes the two hydroxyl groups no longer equivalent. Because of this broken symmetry, we assumed that the tunneling motion evident in the parent species of ethylene glycol was not present in the mono-deuterated species, and that their spectral analyses might be conducted using the standard semirigid *S*-reduced Watson Hamiltonian in its *I*^*r*^ representation.^38^

**Figure 2:**
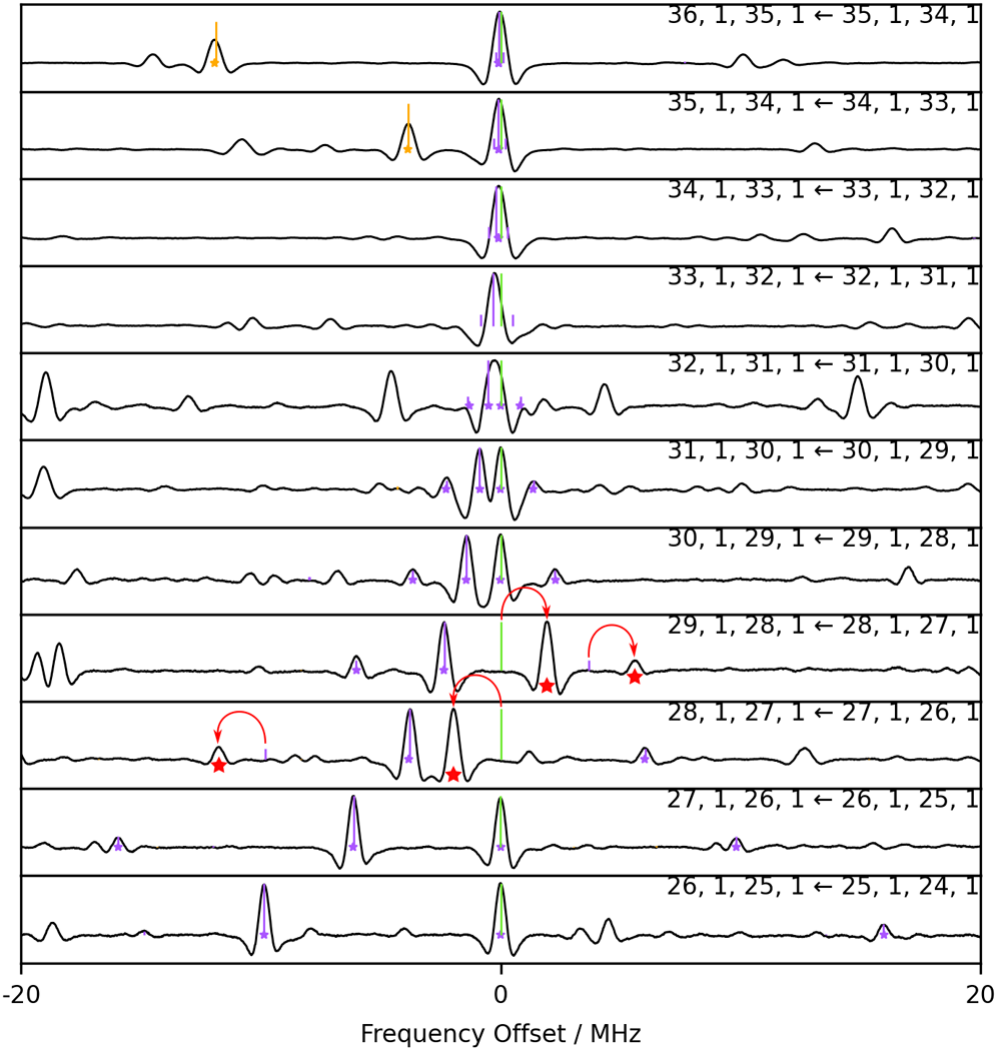
Loomis-Wood plots of OHOD, *aGg*′ conformer. Each plot shows the same spectral pattern, but readers are referred to the third bottommost panel for simplicity: from the left to the right: *b*-type sequence (*K*_a_ = 2 ← 1; violet), *a*-type sequence (*K*_a_ = 2, *K*_a_ + *K*_c_ = *J* + 1; violet), *a*-type sequence (*K*_a_ = 1, *K*_a_ + *K*_c_ = *J*) centered (green lines), *b*-type sequence (*K*_a_ = 1 ← 2; violet). The centered sequence shows some shifted features for *J* = 28 and *J* = 29. Red stars indicate the correct assignment of spectral lines, while red arrows represent unperturbed predictions compared to their correct positions. Orange lines belong to the *aGg*′ species of ODOH.

Incorporation of the newly recorded transitions in the fitting procedure pointed out several perturbed lines (see Figure 2 for an example), which exhibited deviations from our predictions ranging between 0.1 and 10 MHz. The fact that these frequency shifts could not be recovered by adding additional centrifugal distortion terms to the Hamiltonian and that they were observed in both species (more pronounced for OHOD than for ODOH) suggested a possible interaction among them. Indeed, OHOD and ODOH can also be seen as different conformers of the same isotopic species whose interchange occurs through a motion analogous to the tunneling motion taking place in the main isotopic species. The interaction between different conformers of a given molecule has been successfully treated, for instance, in the study of malonaldehyde,^40^ ethanol,^41^ propanol^42^ and deuterated acetaldehyde.^43^ To account for this interaction, both Coriolis and Fermi terms were introduced in the Hamiltonian, together with the energy difference (*E*) between the two mono-deuterated species (see eqs. 2 and 3 of ref (42) for more details about the form of the Hamiltonian). The experimental data were merged into a single dataset. A first guess of *E*, a crucial term for the correct interpretation of the perturbation, was obtained by identifying the most perturbed levels of both species, although this inspection was somehow limited to those levels whose assignments were reasonably secure. After few unsuccessful attempts, the dominant matrix elements of the interaction Hamiltonian (at least at that stage) were found to be those with Δ*K*_*a*_ = 3 and a rough estimate of *E* was obtained by the difference in energy of the 33_4,30_ level of OHOD and the 33_7,27_ level of ODOH.

The initial *E* value was set to approximately 20 cm^–1^, with the ODOH species lying lower in energy than OHOD. This estimate is in agreement, in terms of order of magnitude, with the theoretical evaluation of 11.3 cm^–1^ reported by Boussessi and Senent.^39^ By introducing this energy difference and the *F*_2*ab*_ and *F*_*ab*_ Coriolis coupling constants in the fit, we were able to incorporate tens of perturbed lines in the spectral analysis with residuals in line with the experimental uncertainty. Additionally, spectral predictions based on the newly derived spectroscopic constants showed improved predictive power and allowed us to extend the assignment to higher *K*_*a*_ transitions. As the incorporation of lines proceeded, it was necessary to add further interaction constants in the Hamiltonian, including Coriolis terms of different symmetry, their centrifugal dependence, and a few Fermi constants. The inclusion of these parameters proceeded, in the first place, on the basis of symmetry considerations and in analogy with the set of constants used for the parent species of ethylene glycol; in the second place, we relied on a “trial and error” procedure. The final analysis was based on 4656 *a*- and *b*-type transitions up to *J* = 50 and *K*_*a*_ = 32. In total, 56 distinct parameters were considered to model the spectra of both mono-deuterated *aGg*′ forms between 18 and 450 GHz, successfully reproducing the experimental observations with a root-mean-square error of 41 kHz. For both species, rotational constants, complete quartic and sextic sets of centrifugal distortion constants, as well as a few octic terms, have been determined. The set of spectroscopic parameters also includes five Coriolis coupling constants and their series expansions along with some Fermi terms (*W*_*F*_, *W*_+–_) and their centrifugal dependence. The results of the fit are presented and compared with the calculated and literature values in Table 1, while the list of assigned transitions is provided in the Supporting Information. The energy difference and the interaction parameters, determined for the first time, are instead collected in Table 2.

**Table 1:** Spectroscopic Constants of the Two Mono-Deuterated *aGg*′ Species^a^

		CH_2_OD–CH_2_OH			CH_2_OH–CH_2_OD		
constant	unit	this work (exp)	ref (39) (theo)	ref (28) (exp)	this work (exp)	ref (39) (theo)	ref (28) (exp)
*A*	MHz	14620.246(4)	14621.8	14620.29(1)	15126.688(4)	15140.8	15126.97(3)
*B*	MHz	5548.421(1)	5543.2	5548.453(9)	5311.101(1)	5301.5	5311.05(1)
*C*	MHz	4517.8829(4)	4515	4517.879(9)	4412.1674(4)	4407.5	4412.19(1)
*D*_*J*_	kHz	7.63510(10)	7.615	7.9(2)	6.54498(9)	6.608	6.3(3)
*D*_*JK*_	kHz	–29.679(3)	–25.641	–28.7(2)	–30.495(3)	–28.866	–30.4(4)
*D*_*K*_	kHz	63.044(5)	52.795	52.(2)	80.559(5)	72.202	95.(6)
*d*_1_	kHz	–2.4422(1)	–2.44	–2.474(7)	–2.0259(1)	–2.05	–1.90(1)
*d*_2_	kHz	–0.18059(5)	–0.187	–0.169(3)	–0.14257(4)	–0.144	–0.182(8)
*H*_*J*_	mHz	–13.64(3)	–37.5		–9.51(3)	–26.2	
*H*_*JK*_	mHz	254.1(4)	297		228.1(6)	271.8	
*H*_*KJ*_	Hz	–1.401(2)	–1.0909		–1.559(2)	–1.2892	
*H*_*K*_	Hz	2.148(7)	1.4833		2.803(9)	1.986	
*h*_1_	mHz	–5.38(8)	–17.6		–3.32(8)	–12	
*h*_2_	mHz	0.29(2)	–2.3		0.10(2)	–1.5	
*h*_3_	mHz	0.161(7)	–0.1		0.076(6)	–0.1	
*L*_*JJK*_	μHz				–1.4(2)		
*L*_*JK*_	μHz	–21.1(5)					
*L*_*KKJ*_	μHz	81.(1)			62.(1)		
*l*_1_	μHz	0.07(2)			0.07(2)		

a Notes: Values in parentheses are one standard deviation and refer to the last digits.

**Table 2:** Interaction Parameters and Energy Difference between the Two Mono-Deuterated *aGg*′ Species^a^

constant	unit	value
*E*	cm^–1^	21.74017(2)
*F*_*ab*_	MHz	–1.64(2)
*F*_*ab*,*J*_	kHz	0.763(8)
*F*_*ab*,*K*_	kHz	–3.03(6)
*F*_*ab*,*JK*_	kHz	0.00070(2)
*F*_2*ab*_	kHz	–0.081(1)
*F*_2*ab*,*K*_	Hz	–0.40(2)
*F*_*bc*_	MHz	–0.35(2)
*F*_*bc*,*J*_	kHz	0.143(10)
*F*_*bc*,*K*_	kHz	–0.52(2)
*F*_2*bc*_	kHz	–0.045(3)
*F*_2*bc*,*JK*_	mHz	0.30(5)
*F*_2*ac*_	kHz	–0.158(4)
*F*_2*ac*,*J*_	Hz	0.130(3)
*F*_2*ac*,*K*_	Hz	0.94(5)
*W*_*F*_	cm^–1^	–0.166(7)
*W*_*F*,*J*_	MHz	0.88(6)
*W*_*F*,*JK*_	MHz	–0.0024(1)
*W*_+–_	MHz	0.53(1)
*W*_+–,*K*_	MHz	–0.00023(2)

a Notes: Values in parentheses are one standard deviation and refer to the last digits.

### 3.2 Doubly Deuterated Species—*aGg*′ conformation

As the molecular symmetry of doubly O-deuterated ethylene glycol remains unchanged with respect to the parent species, the effect of the tunneling motion between two equivalent positions of the deuterated hydroxyl groups is well visible in the spectrum.^22,27^ To take this effect into account in the Hamiltonian, the same formalism introduced in previous studies^24,26^ has been used.

The procedure described in the previous section has also been employed for spectral analysis of the doubly deuterated species. In the 75–110 GHz frequency region, the assignment procedure was relatively straightforward, as most of the rotation-inversion lines were found within few MHz from the expected position, e.g., 1 MHz for the *J* = 9–10 transitions. The discrepancy was larger at higher frequency, in the order of tens of MHz for transitions involving levels with *J* > 20, but the correct assignment was always unambiguous up to about 330 GHz. Above this frequency limit, which corresponds to *a*-type transitions involving *J* ≥ 33, it became impossible to follow any sequence of lines even with the use of Loomis-Wood plots, regardless of the chosen *K*_*a*_ value. The underlying reason, which will be discussed in detail in the next section, seems to be the very small energy difference between the pair of inversion states, which results in a huge displacement from the hypothetical unsplit levels. To give an idea, the two tunneling components of the 36_7,30_–35_7,29_ transition are predicted around 640 GHz, while the previous and next *J* of the same sequence are predicted at 340 and 361 GHz, respectively.

Limiting the analysis to the levels that could be modeled successfully, for ODOD, a total of 1707 transitions, which include 1694 *a*- and *b*-type and 13 *x*-type transitions^a^, were assigned between 18 and 332 GHz, with *J* up to 38 and *K*_*a*_ up to 32.

Our model incorporates 32 distinct parameters, which reproduce the experimental features with a root-mean-square error of 43 kHz. The new assignments significantly improve previous results with the full set of sextic centrifugal distortion terms as well as Coriolis coupling constants (*F*_*ab*_ and *F*_*bc*_) and their centrifugal dependencies being additionally determined. Series expansion terms of the energy difference (E*) were also included in the analysis. Results of the fit are reported in Table 3, where they are also shown, compared with calculated and previous experimental values. The list of assigned transitions can be found in the Supporting Information.

**Table 3:** Spectroscopic Parameters Determined for the *aGg*′ Species of Doubly Deuterated Ethylene Glycol^a^

constant	unit	this work (exp)	ref (39) (theo)	ref (22) (exp)	constant	unit	this work (exp)	ref (22) (exp)
*A*	MHz	14380.069(5)	14403.1	14380.07(9)	E*	MHz	293.206(4)	293.20(5)
*B*	MHz	5288.8171(9)	5267.8	5288.81(8)	*E*_J_^*^	kHz	–1.395(8)	
*C*	MHz	4325.1514(7)	4319.3	4325.17(2)	*E*_K_^*^	kHz	–60.28(4)	–69.(4)
*D*_*J*_	kHz	6.9017(5)	6.837	7.2(2)	*E*_JK_^*^	kHz	–0.01355(10)	
*D*_*JK*_	kHz	–30.596(7)	–25.918	–29.6(8)	*E*_JJ_^*^	Hz	–0.082(7)	
*D*_*K*_	kHz	71.6(1)	58.613	69.(3)	*E*_JJK_^*^	Hz	–0.00261(6)	
*d*_1_	kHz	–2.2437(5)	–2.143	–2.2(2)	*E*_2_^*^	Hz	80.(2)	
*d*_2_	kHz	–0.2086(9)	–0.155		*E*_2K_^*^	Hz	–5.7(1)	
*H*_*J*_	mHz	–16.12(7)	–31.2		*E*_2*J*K_^*^	mHz	–0.00301(10)	
*H*_*JK*_	mHz	734.(2)	251.4		*F*_*bc*_	MHz	–37.955(8)	–38.1(1)
*H*_*KJ*_	Hz	–3.082(5)	–1.005		*F*_*bc*,*J*_	MHz	0.00294(1)	
*H*_*K*_	Hz	21.1(6)	1.389		*F*_*bc*,*K*_	MHz	–0.1213(1)	
*h*_1_	mHz	–7.17(8)	–14.6		*F*_2*bc*_	MHz	–0.001465(3)	
*h*_2_	mHz	–2.10(6)	–2.0		*F*_*ab*_	MHz	–358.440(9)	–358.(1)
*h*_3_	mHz	–1.40(3)	–0.1		*F*_*ab*,*J*_	MHz	0.00353(1)	
					*F*_*ab*,*K*_	MHz	–0.01773(5)	
					*F*_2*ab*_	MHz	–0.00482(3)	

a Notes: Values in parentheses denote one standard deviation and apply to the last digits of the constants.

### 3.3 *gGg*′ Conformers

So far, the higher-energy *gGg*′ conformer of ethylene glycol has been identified only for the parent species and the doubly deuterated CD_2_OH–CH_2_OH isotopologue.^29^ According to theoretical calculations,^39^*gGg*′ lies between 45 and 60 cm^–1^ higher in energy (depending on the considered isotopologue) than the corresponding *aGg*′ conformer. This translates into a population, for the *gGg*′ forms, that ranges between 75 and 80% of that of the corresponding *aGg*′ conformers. The calculated energy differences, however, appear to be underestimated when compared with the experimental determination of ref (25), where a value of ∼200 cm^–1^ was derived for the parent species.

After the completion of the spectral analysis of the *aGg*′ mono- and doubly deuterated species, the rotational spectra of their *gGg*′ conformers were predicted using scaled values of the computed spectroscopic parameters.^39^ In particular, the scaled constants were obtained by multiplying the theoretical values for *gGg*′ by the ratio between the experimental and computed ones of the corresponding *aGg*′ form. The assignment and analysis were limited to the 220–450 GHz range, which corresponds to the spectral region that has been recorded continuously (see Section 3).

For the first time, the *gGg*′ conformers of both the OHOD and ODOH species were identified in the spectrum. Their rotational transitions, which involves *J* values above 25 at frequencies higher than 220 GHz, were found at least 150 MHz apart from prediction for OHOD and at least 260 MHz apart in the case of ODOH. Similarly to the *aGg*′ mono-deuterated species, but to a much greater extent, perturbations were clearly observed for several transitions of both forms, even at the lowest *J* and *K*_*a*_ values. Although the same scaling procedure was also applied to the spectroscopic parameters of the *gGg*′ conformer of the doubly deuterated species, no confident assignment of its spectrum was possible. While some sequences of lines seemed to be successfully identified, their analysis resulted in the failure of predicting additional transitions in our spectra. Consequently, we could not confirm the presence of this species.

A total of 517 transitions for ODOH and 323 for OHOD were assigned in the 220–330 GHz range, including *a*-, *b*-, and *c*-type transitions up to *J* = 36 for both species and up to *K*_*a*_ = 13 for ODOH and *K*_*a*_ = 11 for OHOD. The rotational constants together with the full set of quartic and some sextic centrifugal distortion terms were successfully determined for both species. The sextic centrifugal distortion constants that could not be fitted were fixed at the corresponding theoretical values. Our models incorporate 15 and 13 distinct parameters for ODOH and OHOD, respectively, which are able to reproduce the experimental spectrum with a root-mean-square error of 39 kHz and 45 kHz, respectively. A rough estimate of the energy difference between the *aGg*′ and *gGg*′ conformers based on the line intensities observed for several tens of transitions provides a value of ∼ 250 cm^–1^. This is again much larger than the computed value,^39^ but in line with the discrepancy found between theory and experiment for the parent species.

The results of the fit are collected in Table 4 and compared with the corresponding scaled/calculated values, while the list of assigned transitions is available in the Supporting Information. The fit statistics for all of the species studied in this work are summarized in Table 5.

**Table 4:** Spectroscopic Constants of the Two Mono-Deuterated *gGg*′ Species^a^

		CH_2_OD–CH_2_OH			CH_2_OH–CH_2_OD		
constant	unit	this work (exp)	scaled	ref (39) (theo)	this work (exp)	scaled	ref (39) (theo)
*A*	MHz	14492.851(2)	14500.9	14502.4	14704.460(4)	14705.9	14719.7
*B*	MHz	5506.2150(9)	5492.8	5487.6	5319.121(2)	5310.9	5301.3
*C*	MHz	4505.2733(8)	4500.2	4497.3	4472.997(1)	4469.1	4464.4
*D*_*J*_	kHz	7.675(1)	7.751	7.731	6.921(2)	6.977	7.044
*D*_*JK*_	kHz	–29.459(5)	–32.967	–28.482	–29.59(1)	–29.869	–28.273
*D*_*K*_	kHz	61.28(3)	67.30	56.36	72.66(3)	70.856	63.506
*d*_1_	kHz	–2.3900(4)	–2.388	–2.386	–1.9980(8)	–2.038	–2.062
*d*_2_	kHz	–0.1709(2)	–0.163	–0.169	–0.1264(2)	–0.13	–0.13
*H*_*J*_	mHz	–15.3(5)	–13.5	–37.1	–26.(1)	–10.3	–28.5
*H*_*JK*_	mHz	243.(4)	276.9	323.7	246.(9)	272.3	324.5
*H*_*KJ*_	Hz	–1.68(3)	–1.6165	–1.2587	–0.7(1)	–1.7375	–1.4368
*H*_*K*_	Hz	2.1(1)	2.4801	1.7126	3.0115	3.0115	2.1337
*h*_1_	mHz	–5.1(3)	–5.01	–16.4	–9.8(5)	–3.02	–10.9
*h*_2_	mHz	0.6(2)	b	–2.0	–0.9	b	–0.9
*h*_3_	mHz	0.21(6)	b	–0.1	–0.90(7)	b	–0.1

a Notes: Values in parentheses are one standard deviation and refer to the last digits. Parameters without uncertainties are held fixed at the corresponding scaled or theoretical values.

b The *h*_2_ and *h*_3_ constants have not been scaled because of the change in sign from theory to experiment for the *aGg*′ form.

**Table 5:** Fit Statistics

species	transitions	lines	frequency range (GHz)	*J*_max_, *K*_a max_	RMS (kHz)^a^	σ^b^
*aGg*′ – ODOH/OHOD	4656	2996	18–450	50, 32	41.3	1.069
*aGg*′ – ODOD	1707	1160	18–332	38, 32	42.8	0.955
*gGg*′ – ODOH	517	356	221–330	36, 13	39.1	1.565
*gGg*′ – OHOD	323	201	220–328	36, 11	44.8	1.792

a Root-mean-square error of the residuals.

b Weighted standard deviation of the fit.

## 4 Discussion

The analysis of several thousand newly measured rotational transitions for the *aGg*′ conformer of mono- and doubly deuterated ethylene glycol represents a huge improvement with respect to the literature studies,^27,28^ where the dataset for each species was limited to about 50 lines.

As far as the mono-deuterated species are concerned, the spectroscopic parameters derived in this work exhibit uncertainties that are 1 and 2 orders of magnitude smaller than those obtained by Caminati and Corbelli^28^ for the rotational constants and the quartic centrifugal distortion terms, respectively. Additionally, we have determined accurate values for the full set of sextic centrifugal distortion constants and some octic terms, none of which had been previously determined experimentally. Moving to the comparison with theoretical values,^39^ a good agreement is generally observed, with only a few exceptions. While the deviations observed are approximately 0.1% for the rotational constants and a few percent for the quartic centrifugal distortion constants, larger discrepancies are noticed for the sextic terms. Here, the agreement is somehow limited to the sign of the parameters and their order of magnitude with the exclusion of the off-diagonal *h*_2_ and *h*_3_ constants. Nevertheless, similar differences can be observed when comparing theoretical^39^ and experimental^26^ parameters for the main isotopologue of ethylene glycol. Therefore, the problem seems to be more related to the nature of the molecule itself than to the deuterated species.

**Figure 3:**
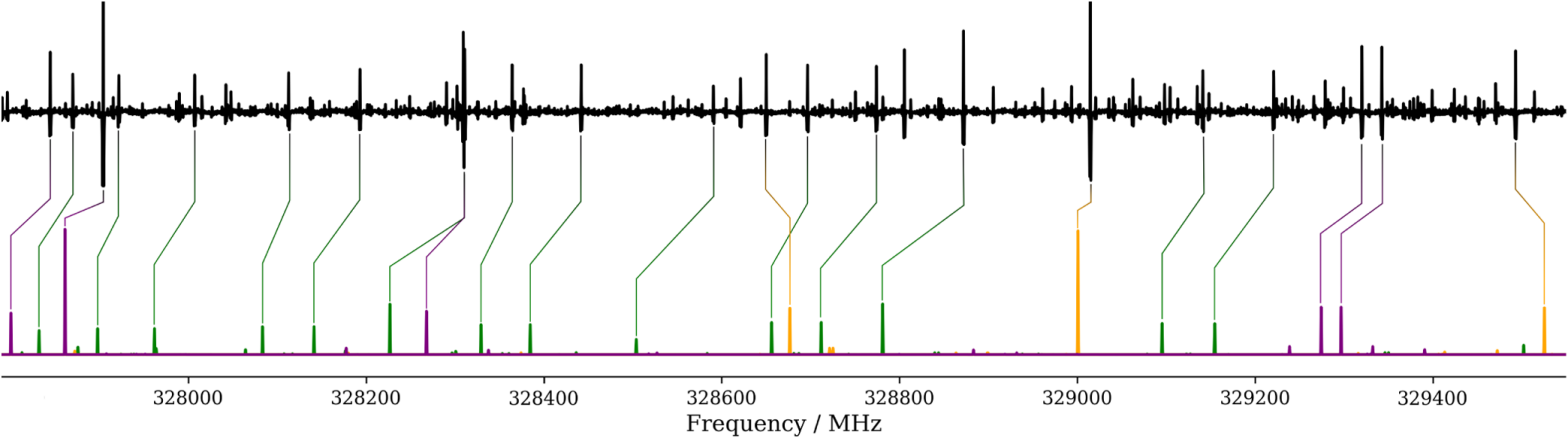
Top: Portion of the millimeter-wave spectrum around 329 GHz. The black trace is the recorded spectrum of the deuterated sample. Bottom: Stick-spectrum predictions of the *aGg*′ species are based on low-frequency data^22,27,28^ (the color legend is the same as Figure 1).

The major achievement obtained in this work is represented by the identification and interpretation of the interaction between the two mono-deuterated species. Inclusion of Coriolis and Fermi constants in the Hamiltonian allowed us to incorporate about 770 transitions that would have been discarded (or not assigned at all) otherwise. This is particularly important when spectroscopic data are used to guide astronomical observations of potentially detectable molecules. In fact, beyond the frequency shift that one could have committed by extrapolating low-frequency data to higher frequencies (see Figure 3), the correct modeling of this interaction is crucial for preventing the misassignment of molecular lines in complex spectral surveys (see Section 6.3 of ref (44) for an example). Despite the large number of spectroscopic parameters needed to model the spectra of the mono-deuterated *aGg*′ species, we expect our analysis to be enough robust to guide astronomical searches of this isotopologue across the whole ALMA Band 7 and very likely also at frequencies close to those covered in our work, thereby including ALMA Band 8 as well.

As a last remark on the *aGg*′ conformers of the mono-deuterated species, it should be highlighted that seven lines assigned by Caminati and Corbelli^28^ to the OHOD species were excluded from our analysis, as they showed deviations from few to tens of MHz with respect to our predictions. While we cannot infer if these lines belong to another species, it is conservative to consider our model as much more robust, so we can cast only some doubt on the assignment of these lines and exclude them from our fit. The inclusion of these lines in the analysis by Caminati and Corbelli^28^ is the reason at the basis of the discrepancy between our and their value for the *D*_*K*_ constant.

**Figure 4:**
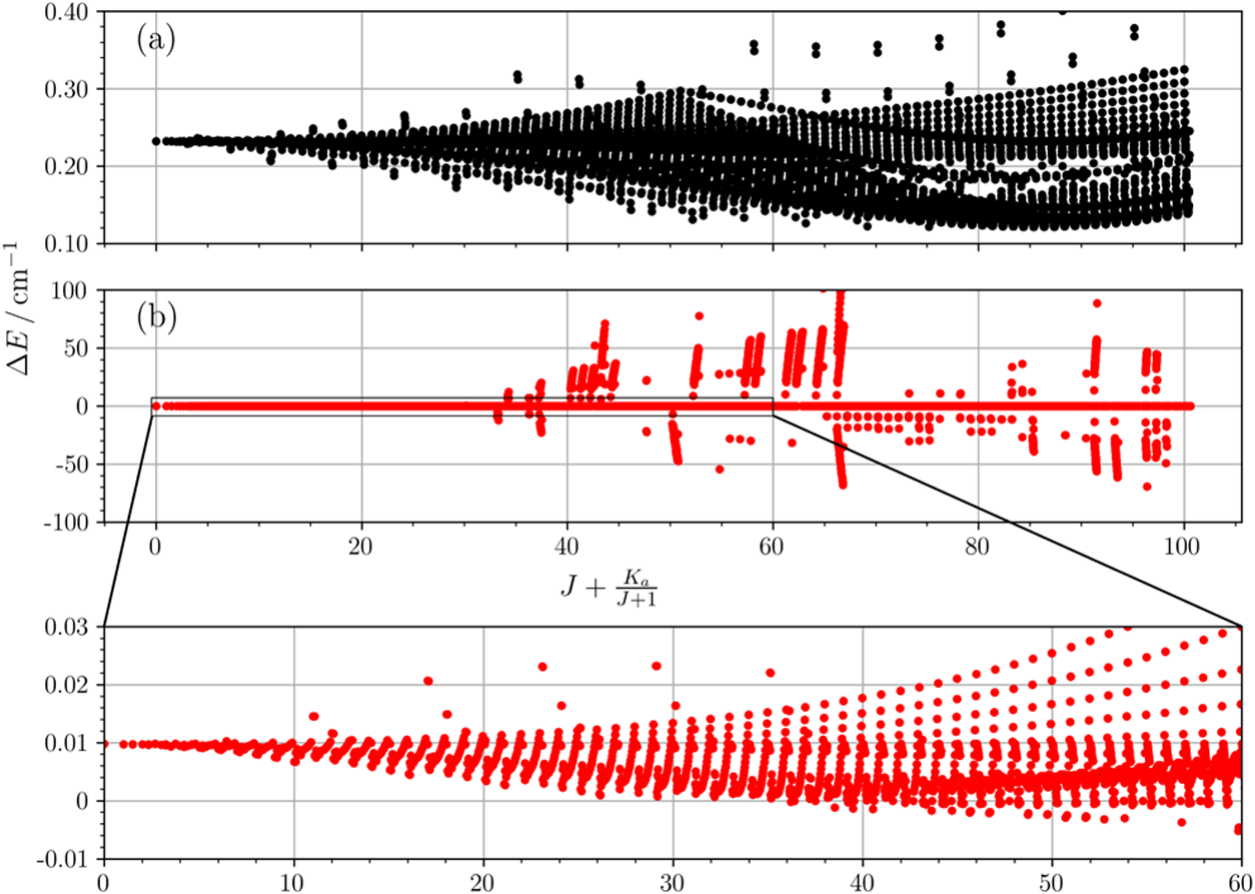
Plots of the energy differences (Δ*E*) between the two tunneling substates as a function of , up to *J* = 100. (a): *aGg*′ conformer of the main isotopologue. (b): *aGg*′ conformer of the ODOD species; a zoom for 0 ≤  ≤ 60 is also provided.

Regarding the doubly deuterated species, similar considerations can be made. The rotational and quartic centrifugal distortion constants are now determined with an accuracy that is 1–3 orders of magnitude better than that of previous works.^22,27^ In addition, more than 20 parameters have been determined for the first time, including the full set of sextic centrifugal distortion terms and several dependencies of both Coriolis coupling constants and the energy difference between the tunneling states. The agreement between the experimental and theoretical data is again good but not excellent, and it worsens while moving to higher-order terms. This discrepancy might be partly related to the difficulties encountered in the assignment of transitions associated with levels with *J* ≥ 33. Above this limit, it became hard to follow almost any sequence of lines, regardless of the *K*_*a*_ values involved. To get insights into this issue, we plotted the tunneling splitting for each *J*_*K*_*a*_, *K*_*c*__ level and made a comparison between the parent and the doubly deuterated species of ethylene glycol (Figure 4). For the parent species, the plot exhibits a smooth trend, with the energy difference remaining around 0.2 cm^–1^ for most levels, and always above 0.1 cm^–1^. In contrast, the doubly deuterated isotopologue shows a breakdown of this pattern beyond *J* = 33, with certain levels deviating up to 100 cm^–1^. Such huge displacements are probably caused by accidental degeneracies among levels, which in turn are the result of the centrifugal dependence of the energy difference between the tunneling substates. At present, our data cannot be safely used to predict rotational transitions above *J* = 33 for this species.

It remains to be investigated whether alternative models can more accurately reproduce the spectrum, even at higher *J* values. The anomalies observed in our model seem to be related with the perturbation method used for describing the interaction between the two tunneling substates, which becomes unphysical whenever their energy difference becomes close to zero. To overcome such limitations, a variational treatment of this interaction or an explicit description of the energy levels based on the dihedral angles associated with the tunneling motion is probably better suited.

A comment on the lines assigned by Walder et al.^27^ is also deserved. As already done by Christen et al.^22^ in their analysis, we discarded the 5_0,5_ (0^+^) ← 4_0,4_ (0^–^) transition from the dataset because of a shift of 5.5 MHz with respect to the predicted value. The transition is most likely misassigned. Moreover, we have corrected the transition frequency of the 5_2,4_ (0^–^) ← 5_3,3_ (0^–^) line from 48,575.4 to 48,574.4 MHz. This re-assignment was suggested in the first place by our improved set of spectroscopic parameters and subsequently confirmed by the value of the tunneling splitting reported in Table 5 of Walder et al.^27^

Another important outcome of our work is the first identification of *gGg*′ conformer of the two mono-deuterated species. This has been made possible by the accurate spectral predictions obtained from the scaling of the computed spectroscopic parameters provided in ref (39). Even though the number of assigned transitions is much lower than that of the *aGg*′ conformers, we could determine several spectroscopic constants with high precision, with the agreement between our experimental values and the theoretical ones being again quite good. The assignment of further transitions was prevented by the strong perturbations observed in all of the targeted sequences of lines. Presumably, the two *gGg*′ species strongly interact with each other (as in the case of the *aGg*′ species), and such interaction heavily affects most energy levels. At present, it is not possible to estimate the energy difference between the two species with reasonable accuracy and, therefore, a combined analysis is not feasible. In this respect, we should emphasize that despite the good agreement observed between experimental and theoretical values, the set of spectroscopic parameters determined for the two *gGg*′ species is highly effective and cannot be used to safely predict rotational transitions that are not included in our analysis. Robust predictions for these species can only be obtained if a correct interpretation of the perturbation is achieved.

As discussed in the previous section, the *gGg*′ conformer of doubly deuterated ethylene glycol could not be confidently assigned in our spectrum. Generally speaking, the *gGg*′ conformers appear to be more difficult to model; whenever difficulties are encountered in the analysis of the *aGg*′ conformers, these become much more pronounced in the corresponding *gGg*′ species.

## 5 Conclusions

The knowledge of the rotational spectra of oxygen-deuterated ethylene glycol, so far limited to low *J* and *K*_*a*_ transitions and restricted to the region below 50 GHz, has been greatly extended owing to the analysis of several thousands of newly assigned transitions and by reaching frequencies as high as 450 GHz. A significant milestone of this work is the identification of Coriolis and Fermi interactions between the *aGg*′ conformers of the singly deuterated CH_2_OH–CH_2_OD and CH_2_OD–CH_2_OH species. The correct modeling of the observed perturbations allowed the accurate determination of the energy difference between these two species, that is, 21.74017(2)cm^–1^, and the incorporation of over 770 transitions in our dataset. Without the correct treatment of Coriolis and Fermi interactions, these lines show significant deviations from the predicted positions. Regarding the doubly deuterated isotopologue in its *aGg*′ form, rotational and centrifugal distortion constants have been refined, and the set of spectroscopic parameters has been greatly expanded. However, the anomalies observed at high frequency values suggest caution in extrapolating spectral predictions for transitions with *J* values of greater than 33. Furthermore, this study reports the first spectroscopic observation of the *gGg*′ mono-deuterated species. Strong perturbations, presumably due to the interaction between the CH_2_OH–CH_2_OD and CH_2_OD–CH_2_OH species, limited the number of assigned transitions, although an accurate determination of several spectroscopic constants up to the sixth order was still possible. No confident assignment could instead be made for the doubly deuterated *gGg*′ species. To conclude, this work provides a robust foundation for future interstellar searches of deuterated forms of ethylene glycol up to the frequency region covered by ALMA Band 7.

## Supporting Information Available

List of files provided (PDF)
Reformatted SPFIT output file for each species analyzed, including all of the assigned transitions and their residual from the final fit (ZIP)

## Footnotes

a The *x*-type transitions observed for this species occur within the same tunneling substate and obey the selection rules Δ*J* = 1, Δ*K*_*a*_ = 0, and Δ*K*_*c*_ = 0,2.
