# Supplementary material for: Deciphering the Complexity in the Rotational Spectrum of Deuterated Ethylene Glycol
Source: ACS Earth Space Chem. 2025 Apr 29;9(5):1267–76. doi: 10.1021/acsearthspacechem.5c00067 (PMC12086960; doi:10.1021/acsearthspacechem.5c00067)
Supplement: Supplementary file 1 — sp5c00067_si_001.pdf [file sp5c00067_si_001.pdf]

Supporting Information: Deciphering the Complexity in the Rotational Spectrum of Deuterated Ethylene Glycol

Jordan A. Claus,[†] Mattia Melosso,[*,†] Agathe Maillard,[†,¶] Luca Bizzocchi,[†] Vincenzo Barone,[‡] and Cristina Puzzarini[†]

†*Dipartimento di Chimica "Giacomo Ciamician", Università di Bologna, Via F. Selmi 2, 40126 Bologna, Italy*

‡*INSTM, 50121 Firenze, Italy*

¶*Current address: PhLAM – Physique des Lasers, Atomes et Molécules, Université de Lille, UMR 8523 – F-59000 Lille, France*

E-mail: mattia.melosso2@unibo.it

1 List of files provided

The following files are provided as Supporting Information:

1. `ODOH_OHOD_aGg.pi`: combined fit of the *aGg′* conformer of the mono-deuterated species CH_2OD-CH_2OH and CH_2OH-CH_2OD;
2. `ODOD_aGg.pi`: fit of the *aGg′* conformer of the doubly-deuterated species CH_2OD-CH_2OD;
3. `ODOH_gGg.pi`: fit of the *gGg′* conformer of the mono-deuterated species CH_2OD-CH_2OH;
4. `OHOD_gGg.pi`: fit of the *gGg′* conformer of the mono-deuterated species CH_2OD-CH_2OH.

Each file consists of a reformatted version of the standard SPFIT output file (*.fit) using the PIFORM code available on the PROSPE website (Programs for ROtational SPEctroscopy, `http://info.ifpan.edu.pl/~kisiel/asym/asym.htm\#piform`)

The files are composed by several blocks containing:

- The list of assigned transitions

  Each line contains quantum numbers for the upper and lower states (J', K'_a, K'_c, v', J, K_a, K_c, v; see below for more details about the meaning of v), observed frequency in MHz, difference between observed and calculated frequency (obs.-calc.) in MHz, measurement uncertainty in MHz, and two optional columns in case of blended lines (obs.-calc. value with respect to the frequency of the blend and the relative weight of line within the blend);
- Fitted parameters with their uncertainties;
- Fit statistics, such as the root-mean-square (rms) error, the weighted rms, the number of distinct parameters and lines in the fit, and so on;
- Fitted parameters with their standard errors;
- A list of worst fitted constants;
- Correlations between parameters;
- A list of largest correlation coefficients;
- A list of worst fitting lines.

The v quantum number has the following meaning:

1. `ODOH_OHOD_aGg.pi`: $v = 0$ stands for the ODOH species and $v = 1$ for the OHOD species;
2. `ODOD_aGg.pi`: v refers to the tunnelling substate which can take the value of 0 or 1, with the $v = 1$ state being higher in energy than the $v = 0$ state;
3. `ODOH_gGg.pi` and `OHOD_gGg.pi`: no use of v;
